# Understanding dance/movement therapy: a qualitative study of Chinese parents of children with autism spectrum disorder

**DOI:** 10.3389/fpsyg.2025.1616329

**Published:** 2025-11-04

**Authors:** Xing Fan, Kyung Soon Ko

**Affiliations:** ^1^College of Preschool Education, Capital Normal University, Beijing, China; ^2^Department Creative Arts Psychotherapy, Jeonju University, Jeonju, Republic of Korea

**Keywords:** dance/movement therapy, autism spectrum disorder, caregivers, parental perception, China

## Abstract

**Introduction:**

This study explored the application of dance/movement therapy (DMT), a therapeutic method originating from the West, in the treatment of children with autism spectrum disorder (ASD) in China. Specifically, it examined how these children’s parents understood and perceived DMT.

**Methods:**

A descriptive qualitative research design was employed. Five Chinese parents of children with ASD participated. Data were collected through three sources: videos of sessions, in-depth interviews, and movement interviews. Data analysis involved coding and categorization, resulting in 158 codes, 48 subcategories, and 14 categories.

**Results:**

Five main themes were identified: (1) Facing the Unknown but Willing to Try, (2) Observing Embodied Communication and Emotional Growth, (3) Witnessing an Unexpected Therapeutic Relationship, (4) Seeing Therapy Extend into Daily Life, and (5) Reflecting on Parenting and Family Changes.

**Discussion:**

The findings provide foundational insights into how parents of children with ASD perceive DMT in the Chinese cultural context. These results highlight the potential for implementing and promoting DMT in China based on parental perspectives.

## 1 Introduction

Autism spectrum disorder (ASD) is a neurodevelopmental disorder characterized by deficits in social interaction and communication, alongside the presence of repetitive behaviors (American Psychiatric Association, 2013). However, comprehensive understanding of ASD necessitates moving beyond a deficit-centered model and incorporating a strengths-based perspective. Individuals with ASD exhibit considerable heterogeneity and often possess unique strengths, such as exceptional attention to detail and deep, sustained focus on areas of interest (Baron-Cohen et al., 2009). Research indicates that children with ASD demonstrate non-linear developmental trajectories, which evolve with accumulated experience, increased maturation, and enhanced support (South, 2020). The global prevalence of ASD is increasing, and has reached 2.76% among 8-year-old children in the United States (Maenner et al., 2023). However, lack of nationwide surveys hampers an accurate prevalence rate in China. China’s 2020 census reported over 300 million children under 14 years old. Applying the 1–2% global prevalence, approximately 3–6 million children in China may have ASD, which places significant emotional and practical burdens on their parents (China Women and Children Health Association, 2021; Jia, 2023). Therefore, at least 3 million parents of children with ASD are experiencing significant pressure and challenges in their work and daily lives in China (China Philanthropy Research Institute, 2024).

To address the challenges of ASD, the Chinese government introduced a series of policies and assistance measures at the national level. Furthermore, the National Health Commission and other departments have jointly formulated the *National Mental Health Work Plan (2015–2020)* (National Health and Family Planning Commission of the PRC, 2015), which targets children with ASD for prevention and treatment (China Philanthropy Research Institute, 2024). In 2022, the National Health Commission issued the *Standards for Autism Screening and Intervention Services for Children Aged 0–6 (Trial)*, which aims to standardize screening, diagnosis, and treatment for children with ASD to improve their prognosis (National Health Commission of the PRC, 2024). These treatments follow behavioral learning principles.

Dance/movement therapy (DMT) is a psychotherapeutic approach that integrates movement and emotional expression to promote holistic development (American Dance Therapy Association (ADTA), 2024; Meekums, 2003). DMT has been systematically developed, and is academically recognized and widely applied in the West, which aligns with the Eastern philosophy of the unity of mind and body. This philosophy has been an important foundation for mental health and daily life for thousands of years in the East. DMT combines modern psychology and dance, and requires systematic and professional education and training before being applied to clinical treatment.

To integrate DMT into the local Chinese culture and provide high-quality clinical services for children in need, comprehensively understanding parents’ perspectives on DMT is imperative. This study aimed to explore the understanding and perception of DMT from the perspective of Chinese parents of children with ASD. Therefore, we proposed the following research questions:

1. What experiences do parents of children with ASD gain through their children’s participation in DMT?
2. What significance does dance movement therapy (DMT) hold for the parents of children with ASD?

### 1.1 ASD and DMT in China

ASD is a neurodevelopmental disorder (American Psychiatric Association, 2013). The Diagnostic and Statistical Manual of Mental Disorders, Fifth Edition (DSM-5) provides specific criteria for the diagnosis of ASD: (1) persistent deficits exist in social communication and interaction, (2) restricted and repetitive behavior patterns, interests, or activities, (3) symptoms typically occurring in early childhood, (4) symptoms leading to severe impairments in social, occupational, or other important functional areas, and (5) impairments cannot be better explained solely by intellectual disability or overall developmental delay (American Psychiatric Association, 2013).

DMT, a discipline of psychotherapy, uses human movement as a core concept to promote individuals’ comprehensive development (Dance & Arts Therapy, 2024; Devereaux, 2017; Meekums, 2003). DMT is known as dance movement psychotherapy in the United Kingdom, and practitioners regard the body as a communication tool with expressive power and rich connotations. It helps individuals improve emotionally, cognitively, physically, socially, and spiritually through in-depth interactions with their therapists (ADMPUK, 2024).

Previous studies have revealed that DMT alleviates emotional and behavioral problems (Chu et al., 2015; Morris et al., 2021) and improves language expression and cognitive development in children with ASD (Fan and Ko, 2023; Scharoun et al., 2014). Furthermore, it positively impacts the promotion of social interaction, a core difference between children with ASD and neurotypical development (Cui and Wang, 2024; Fitzpatrick, 2018; Millar, 2023). However, DMT research in China has mainly focused on theoretical reviews (Li, 2014; Lin, 2018; Zhou, 2016) and evaluations of clinical-efficacy (Wang et al., 2023; Yu et al., 2020; Zou et al., 2020); feedback on DMT from the perspective of parents of children with ASD is lacking.

## 2 Methods

This study adopted a descriptive qualitative study method to explore how DMT was understood by Chinese parents of children with ASD. Qualitative research aimed to verify objective facts and follow the constructivist paradigm, and focused on in-depth exploration of human-life experiences to obtain a rich and profound understanding (Creswell, 2013). Previous descriptive qualitative studies demonstrated its applicability across various life experiences and the health domain. Previous research explored various themes, such as the need of support from parents of children with rare diseases (Pelentsov et al., 2016) and experience of parents who lost loved ones due to dementia (Peacock et al., 2014). These studies illustrated how the descriptive qualitative approach effectively captured personal and interpersonal experiences during significant life events. In alignment with this established research tradition, we employed a descriptive qualitative design to richly and directly represent participants’ nuanced understanding and perspectives.

### 2.1 Participants

Purposeful sampling was used to select participants who could provide rich, relevant insights aligned with the research objectives (Suzuki et al., 2007). Finally, three fathers and two mothers were recruited; parents maintained prolonged observational engagement with the researcher throughout the one-on-one DMT sessions delivered to their children.

All participants provided informed consent and voluntarily agreed to participate. Inclusion criteria were (1) caregiver of a child diagnosed with ASD, (2) children with ASD had received DMT for >6 months, and (3) caregivers consented to the recording of their children’s DMT sessions and provision of video materials for research purposes.

All participants used pseudonyms to ensure anonymity. They were residents of Beijing, China, and aged 37–47 years. Each had only one child, except for participant Fan who had two daughters. All five children, aged 4–8 years, were diagnosed with ASD at approximately 3 years of age. Their behavioral characteristics exhibited significant heterogeneity: C1 displayed narrow interests and stereotyped behaviors; both C2 and C5 exhibited pronounced social anxiety, and C5 also experienced language regression; C3 and C4 shared difficulties with emotional regulation, which manifested as excessively heightened and uncontrollable emotional expressions. At the time of the study, the children had received at least 7 months of DMT, with the longest course of treatment being 4 years. Table 1 presents participating parents’ basic information.

**Table 1:** Participants’ basic information (DMT intervention for children with ASD).

Pseudonym	Age/Sex	Educational background	Profession	Child (Code)	Child age/Sex
Wang	37/M	PhD	University Professor	C1	7/Boy
Zhang	37/F	MA	Head of Bank Department	C2	7/Boy
Lin	39/M	BA	Freelancer	C3	4/Girl
Chen	39/F	PhD	University Lecturer	C4	4/Girl
Fan	47/M	BA	Enterprise Employee	C5	8/Girl

Different DMT methods were used to treat the five children with ASD, tailored to their individual characteristics and needs. Therapists primarily employed mirroring and attunement techniques (Adler, 1968, 1970; Samaritter and Payne, 2017; Takahashi et al., 2019) to support non-verbal social interaction. Therapists established a sense of connection and trust with the children through movement imitation, sound synchronization, and emotional attunement, which effectively regulated shared emotional states (Manders et al., 2022). Accordingly, new movements were introduced to expand the children’s movement repertoire, and various props were utilized to maintain engagement (Fan and Ko, 2023; Levy, 1992) and achieve specific therapeutic goals. Preferred objects were used to facilitate children’s visual tracking and enhance attention; dolls served as proxies for role-playing and emotional projection; furthermore, building blocks were employed to cultivate an awareness of turn-taking and cooperative play.

### 2.2 Data types

Three types of qualitative data were collected: videos of the sessions, in-depth interviews, and movement interviews. Qualitative studies integrate multiple data-collection methods into a single research project, a widely adopted and effective practice (Moser and Korstjens, 2018) to enhance the credibility and validity of the results.

#### 2.2.1 Videos of the sessions

DMT video data of the children with ASD were collected with the consent of their parents. These recordings vividly captured children’s behavioral expressions, characteristics, and changes during the DMT process. Before the interviews, researchers shared three selected recordings and asked the participating parents to watch them and identify meaningful segments of DMT sessions conducted with their children. During the interviews, participants reviewed these videos and paid particular attention to the following aspects: (1) the regulation of children’s emotions, (2) interactions with therapists, and (3) emotional exchanges generally lacking in daily parent–child interactions. Their focus on (1) and (2) directly stemmed from their urgent expectation for tangible symptom improvement and therapeutic efficacy. In contrast, their deep valuation of (3) revealed a core desire to establish a reciprocal “giving and receiving” emotional relationship with their child.

#### 2.2.2 Interviews

Semi-structured interviews comprised questions aimed to clarify and delve into the research topic yet provided the researchers and interviewees with ample flexibility to have rich and expansive conversations (Dieleman et al., 2018; Reddy et al., 2019; Vanaken et al., 2024). Specific questions from 13 questions included: (1) What scene in the video touched you the most? (2) Which activity left a deep impression on you? Please provide your reasons. (3) How do you now view your child’s physical expressions? Has there been any change in your understanding? (4) What kind of help do you think dance/movement therapy has provided to your child? Each interview was conducted in Chinese, recorded, and lasted approximately 60–110 min.

#### 2.2.3 Movement interviews

In DMT, movement is considered as a powerful communication medium that conveys information far beyond the scope of words alone (Levy, 1992). Hence, movement is also an important type of data in qualitative studies. Therefore, data collection should not be limited to verbal statements, and obtaining participants’ movement data was equally important. The researcher posed the following guiding question to the participating parents: “*If you were to respond to your child in the video with body movements, what movement would you use for expression*?” Among the five participants, four were actively willing to participate in movement expression, while one was not. Responses of the four participants were all spontaneously generated and based on kinesthetic empathy, rather than predetermined gestures or postures. Guided by the interviewer, they translated their emotional perceptions of the video content into non-verbal actions to establish a connection with their children. Among them, two parents crossed their arms and attempted to create a safe boundary for their children using their bodies as protective containers. One parent adopted a gesture of bent elbows and clenched fists, which intended to convey confidence in facing ASD and provide an anchor of strength for the child. Another parent used gentle, tentative fingertip touches, and clarified: “*I want to get closer, but I am afraid of being rejected*.”

### 2.3 Data analysis

Data were sourced from three components: (a) session videos, (b) interviews, and (c) movement interviews. All language data, which included verbal feedback elicited by the videos and content from the semi-structured interviews, were recorded and transcribed via Clova Notes (Line WORKS Corp, 2024). Researchers repeatedly compared and verified the transcripts against the original recordings to ensure transcription accuracy. After transcription, all texts were imported into the qualitative data analysis software MAXQDA (Verbi, 2022) for processing. Data analysis followed the thematic analysis framework proposed by Creswell (2014) and Moustakas (1994), with the specific steps: (1) repeated reading of the transcribed texts, (2) multiple rounds of coding and thematic categorization, which initially generated 158 initial codes through open coding, subsequently condensed them into 48 subcategories and further refined these into 18 categories via axial coding, (3) extraction of final themes upon reaching theoretical saturation, and (4) tabulation of the data and interpreting the results. Movement interviews, which served as an independent and complementary data source, were documented in photographic form and systematically described based on Laban Movement Analysis theory (Wahl, 2019). This data primarily provided experiential support from a bodily perspective for the arguments in Theme 5, which focused on participants’ embodied experiences.

All data analyses were conducted in the original language (Chinese) by Chinese researchers to ensure semantic and cultural contextual accuracy. Translation process involved translation, proofreading, and joint confirmation to ensure that the academic expressions were accurate and consistent in both Chinese and English.

### 2.4 Validity

#### 2.4.1 Participant checks

Researchers returned the transcribed texts of the interview recordings and results to the participants for personal review (Birt et al., 2016), which ensured the transcription was faithful to the original conversation. During feedback, Wang requested the deletion of all content related to “family member relationships,” while Lin requested the deletion of any content not directly related to this study. Researchers respectfully responded to these requests and excluded the related content.

#### 2.4.2 Peer debriefing

In qualitative studies, peer checking is a collaborative process in which the researchers share and discuss research results, understanding, and methods with their peers. This process promotes the development of critical dialogue, which helps researchers gain new insights and share them (Janesick, 2007; Lincoln and Guba, 1985; Spall, 1998). We invited two peer reviewers to conduct a comprehensive review of the data-analysis process (including coding, category, and topic export) and provide feedback to ensure the objectivity and accuracy of the analysis results. Peer reviewers carefully examined the researchers’ ideas and feelings to comprehensively and objectively analyze the data.

### 2.5 Ethical considerations

This study is part of a doctoral dissertation (Fan, 2025), and was approved by the Jeonju university institutional review board (JJ IRB-230503-HR-2023-0406). The researcher provided participants with a detailed explanation of the research procedures and methods, research objectives and significance, right to withdraw from the study, assured them of confidentiality and anonymity measures, data-usage principles, personal-information protection systems, as well as filming and recording. Information was orally presented, and each participant subsequently signed a written informed consent form. In addition, all data was stored in the researcher’s dedicated encrypted personal computer and external hard drive to ensure data security and privacy.

## 3 Results

After the data analysis, 158 codes, 48 subcategories, 14 categories, and five themes were exported. Table 2 presents the analysis results.

**Table 2:** Data-analysis results: Themes and categories.

Theme (5)	Category (14)
Theme 1: Facing the unknown but willing to try	Lacking information about DMT
	Willing to try DMT
Theme 2: Observing embodied communication and emotional growth	Embodied therapeutic communication
	Surprising new responses and interactions
	Growth in verbal and nonverbal communication
	A joyful body and stabilized emotions
Theme 3: Witnessing an unexpected therapeutic relationship	Facilitating participation through movement
	Acceptance catalyzes children’s expressions
	Trusting relationships and investment in movement
Theme 4: Seeing therapy extend into daily life	From therapy to daily physical interaction
	Developing self and daily social interaction
Theme 5: Reflecting on parenting and family changes	Parental self-reflection and emotional awareness
	Transforming parenting and family relationships
	Recognizing the value and future of DMT

This framework comprises five core findings: (a) coexistence of high acceptance and low awareness of DMT among the parents, (b) body’s role as the primary agent of communication and development, (c) therapists as catalytic agents, (d) transference of therapeutic effects to daily life, and (e) transformation of family relationships and parenting styles. Sub-categories are excluded due to space limitations.

### 3.1 Theme 1: Facing the unknown but willing to try

#### 3.1.1 Category 1: Lacking information about DMT

Most participants had difficulty accessing relevant resources or information on DMT. Many participants mistakenly equated DMT with dance classes. Lin and Chen stated that China lacked official DMT information, which made it difficult to learn through formal channels. They mainly relied on social networking communities for information. Chen said, “*I envy the United States for having professional journals in DMT*.” Fan admitted, “*At first, I mistakenly thought that DMT was a dance class.*” Zhang said, “*DMT is undoubtedly closely related to dance or music classes, and its essence is more inclined toward art education*.” Lin said, “*It is probably mainly focused on dance teaching, helping children release negative emotions through dance*.”

#### 3.1.2 Category 2: willing to try DMT

Participants were actively looking for methods to aid their children’s rehabilitation. Therefore, they were open to any method that might help with ASD. This led them to try DMT, still unfamiliar in China. Wang said, “*As parents, we are willing to try more different treatment methods. As it is called DMT, we at least believe that it is harmless to our child*.” Zhang recalled, “*I was not sure what DMT was, but after confirming that it would not cause psychological trauma to my child, I decided to try it*.” Their willingness reflected both hope for recovery and a strong sense of responsibility. This may be related to traditional Chinese family values. Hence, children’s health was related to personal well-being and carried the important significance of parents’ expectations, maintaining family prosperity, and continuity.

### 3.2 Theme 2: Observing embodied communication and emotional growth

#### 3.2.1 Category 1: Embodied therapeutic communication

DMT employs movement as a primary medium to facilitate communication between the inner world of children with ASD and their social environment. Hence, emotions often locked within these children can be expressed and shared through intentional movement. As Zhang noted,

*I can divide the current treatments into two categories: technique and psychological courses. Technique training is certainly important for children, but it is not everything. With its skillful integration, DMT closely combines skills with psychological support and touches on children’s spiritual and emotional needs.*

In addition, most parents noticed that DMT emphasized guiding from the child’s current emotional and psychological state, and witnessed how DMT allowed their children to express themselves freely through body movement during dance. Chen highlighted that, “*This treatment not only provides a healthy and positive emotional outlet for children, promoting their inner harmony and balance, but also teaches them to manage and express themselves more socially and appropriately*.” Parents realized that movement was both an external expression of children’s inner experiences and important bridge to establishing connections with the external world.

Body language emerged as a crucial element in social development. Through DMT, the parents noticed that their children could accurately convey needs, desires, and feelings non-verbally using gestures, facial expressions, and body posture. Chen explained, “*for children, body language often played a more dominant role than verbal language in interactions*.” She explained, “*When children participate in games, they do not always rely on language. Even if they just stand aside, their body posture and facial expressions can clearly convey their desire to participate.*”

Many parents also reported that DMT enhanced their understanding of the body as a powerful expressive tool. They recognized that the body possesses a unique capacity for communication, even in the absence of speech. Wang commented,

*Although I do not understand art, art therapy can indeed interpret an individual’s inner thoughts from the perspective of body language. Body language is different from spoken language. As we all know, dance, as an art of the body, is a vivid manifestation of body language*.

Lin further added,

*Body language is like eye contact, and a lack of eye contact can lead to a lack of emotional tone in communication. For children who already lack interaction, attention, movement, and language abilities, if their bodies were stiff or motionless, their whole person would appear particularly rigid and dull. This state might exacerbate their limited communication with the external world and also affect their understanding of external information and feedback*.

Parents emphasized the body’s significance as a means of communication and gained a deeper appreciation of how movement serves as a foundation for social communication and development. Although DMT is not yet widely practiced in China, these experiences suggest its potential as an effective therapeutic tool for enhancing communication and expression among children with ASD.

#### 3.2.2 Category 2: Surprising new responses and interactions

**Figure 1:**
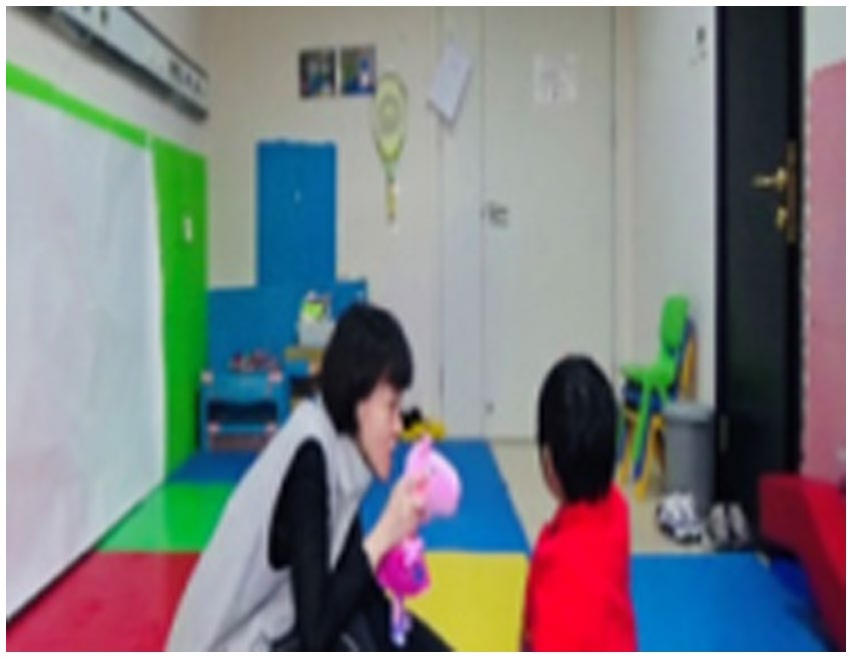
A child who has no interest at all in the therapist. The therapist tried to capture the child's interest by holding a Peppa Pig toy, which the child likes, but no matter what the therapist said or did, the child always looked elsewhere, seemingly uninterested.

**Figure 2:**
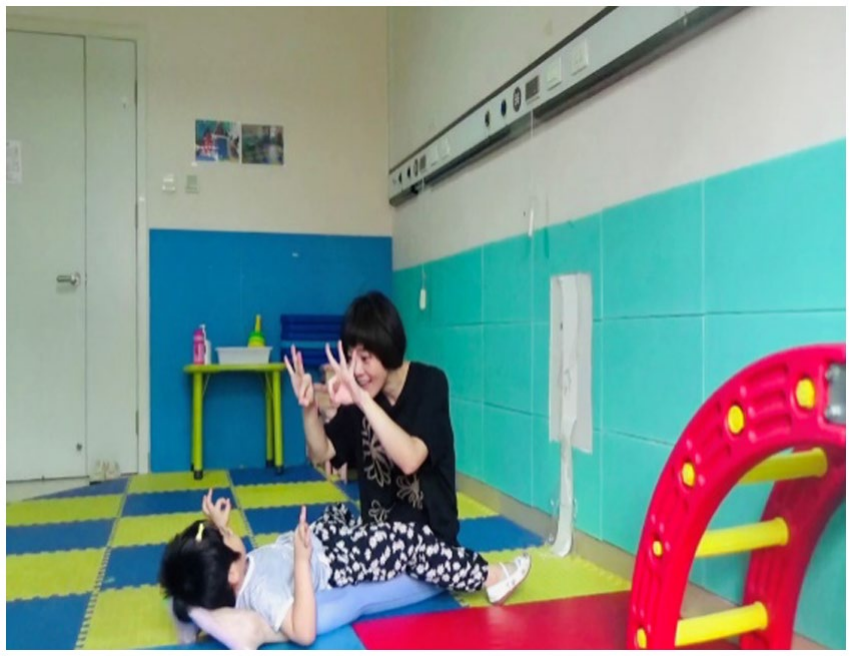
Mirroring movements with the therapist. At this time, the child was lying freely and comfortably on the therapist's lap, mirroring the therapist's movements.

In the recordings, parents were surprised and delighted to witness the effective and positive interactive behaviors exhibited by their children. Fan shared his observation (see Figures 1, 2):

*In the first video, there was very little interaction between my child and the therapist, and she was completely immersed in her own world, talking to herself. However, in the third video, my child showed a surprising level of imitation that far exceeded my expectations in terms of interaction with others*.

**Figure 3:**
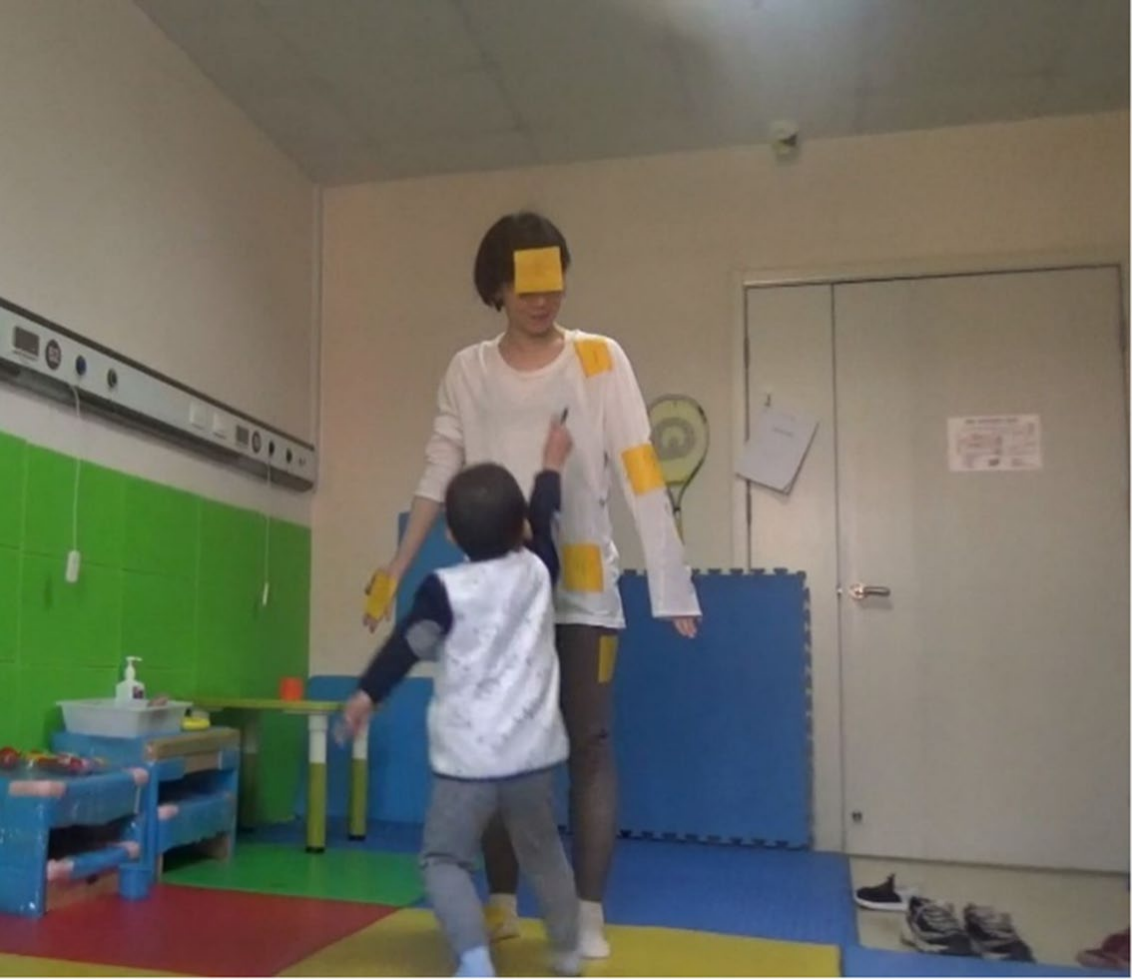
A child playing a body number game with the therapist.

**Figure 4:**
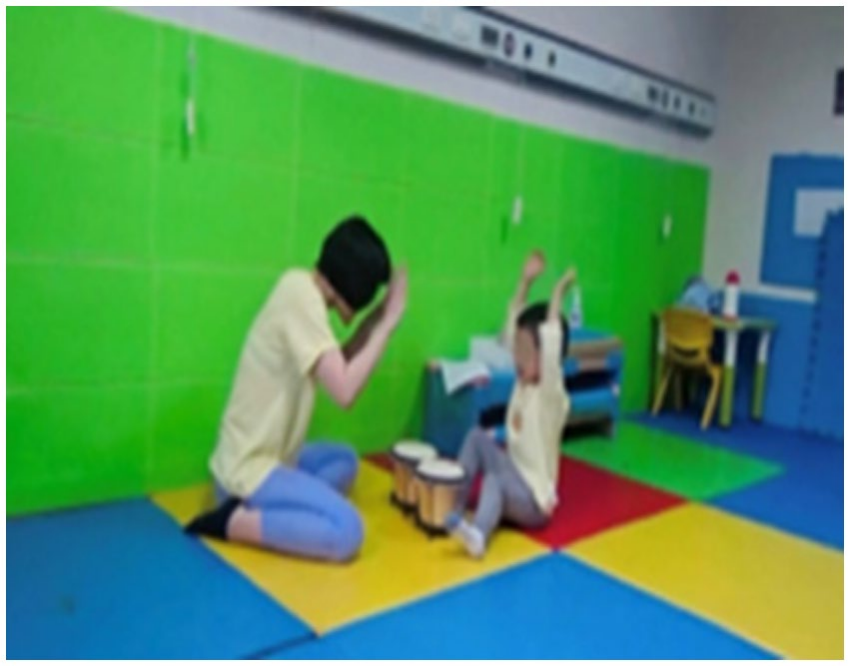
A child playing the drum with the therapist. The child enjoyed beating the drum with both hands alongside the therapist.

Wang recalled:

*The first video was a game about body numbers. My child followed you to identify the numbers. In the second video, you played the drums together, and my child imitated your movements, with your rhythms matching each other. These images are very heartwarming, and he was willing to follow you as the therapist, which means he was focused on participating in the current activity* (see Figures 3, 4).

**Figure 5:**
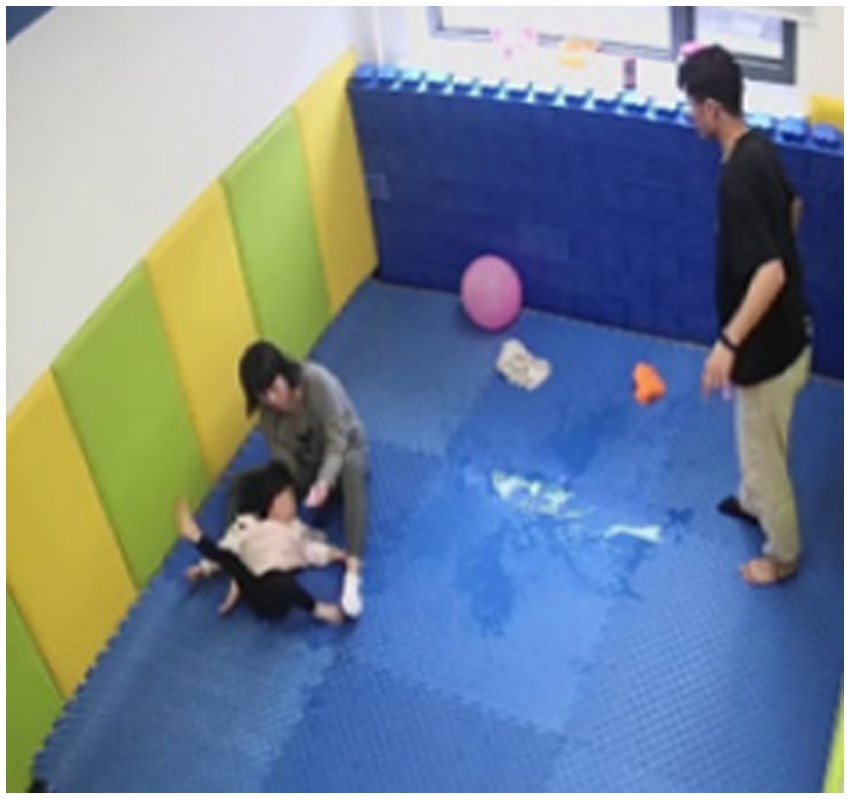
A child in an emotional outburst. In the picture, you can see water spilling and the child's intense emotional expression. The child was lying on the floor, highly agitated, punching and kicking.

**Figure 6:**
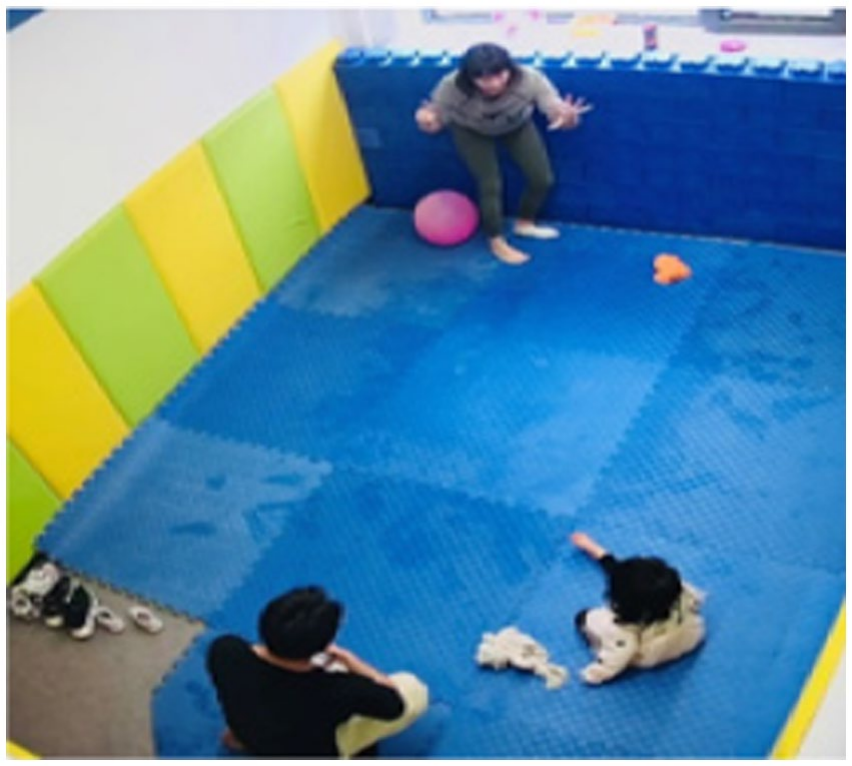
A child capable of communicating with the therapist even during an emotional outburst. The picture on the right showed a scene where this child could follow the therapist's instructions even while experiencing an emotional outburst. The therapist extended five fingers on the left hand to indicate "big" and held up only the index finger on the right hand to indicate "small," guiding the child's choice.

Chen shared, with excitement:

*From the 31st second of the first video, I witnessed a scene of my child’s emotional outburst. She obediently counted and waited in order to get the ball. This behavior was challenging in the context of that time. You know, at that time, my child’s emotional problems were quite serious, and such following and cooperation surprised me* (see Figures 5, 6).

**Figure 7:**
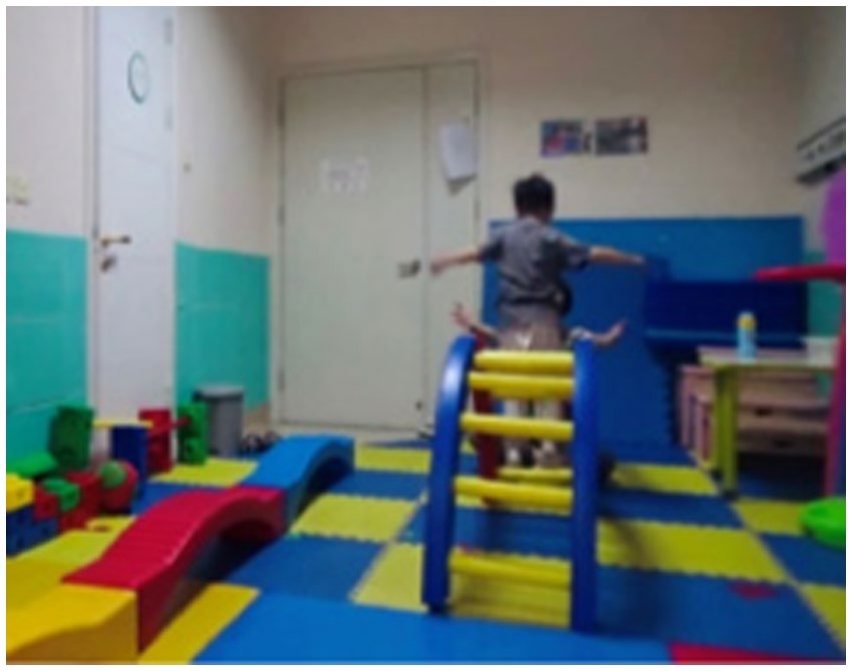
A child with arms open to the therapist. What can be clearly seen in the picture on the left is the moment when the child spreads their arms wide and runs to the therapist immediately upon getting off the jungle gym.

**Figure 8:**
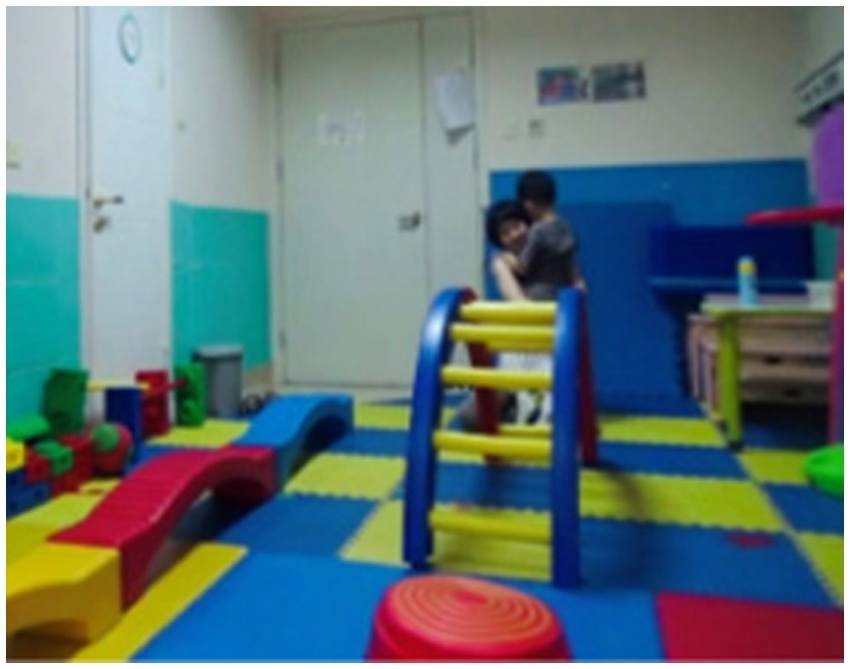
A child embraced in the therapist’s arms. The picture on the right shows the child running into the therapist's embrace.

In addition, parents also commented on the intimate and harmonious interactions between their children and therapist. Zhang said, “*I am very... jealous! My child has never rushed toward me without hesitation and hugged me like this*.” (see Figures 7, 8).

Fan also recalled a similar scene:

*The scene of my child lying peacefully on your lap and having a conversation deeply touched me. At home, if my child had similar behavior, I would often push her away or scold her; however, you chose to accept and continue to interact with my child, which surprised me. Based on this warmth and acceptance, my child seemed more willing to express herself*.

Parents were surprised by the positive interactions and behavioral changes between their children and therapists, and gradually realized that the potential for these positive changes might have already been hidden in their children but had not received sufficient attention.

#### 3.2.3 Category 3: Growth in verbal and nonverbal communication

**Figure 9:**
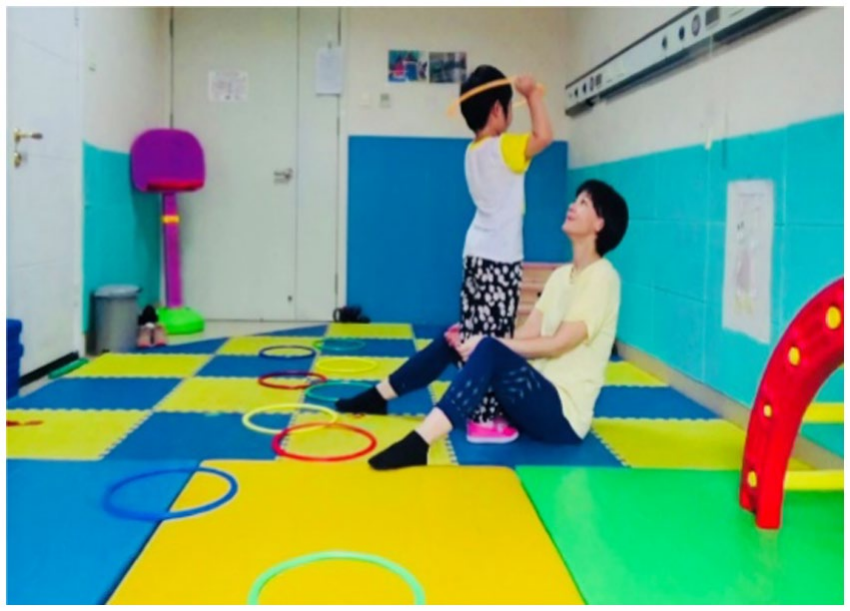
A child playfully interacting with the therapist as witnessed by Fan. When the child and the therapist were engaged in a color differentiation activity, the child playfully placed an orange ring on their head and said with a smile, "This is skin color."

All the parents observed notable advancements in their children’s communication skills, which encompassed both non-verbal expression and verbal language. This progress was often vividly described as an unlocking of potential. Fan and Zhang compared the therapeutic process to a rusty faucet being turned on, which allowed water (language) to gush out freely. Fan was surprised by his child’s lively behavior:

*In the video, the two of you were identifying colors, and my child mischievously smiled and said, “This is skin color.” This playful interaction is what a child should look like. What gratifies me most is that our conversations are now reciprocal; she responds, and I understand her* (see Figure 9).

Similarly, Chen reported a shift from passive response to active inquiry: “*My child has begun to initiate questions like ‘Who should I give the fox to?’ and ‘Where can I watch the performance?*’” This burgeoning verbal ability allowed for more complex social interaction, as Zhang noted “*Nowadays, my child uses words to play tricks and manipulate the nanny*,” which demonstrated an understanding of social dynamics.

A key insight from parents was that the development of non-verbal and verbal communication was deeply intertwined. They observed that an increase in non-verbal expressions, such as gestures, was closely correlated with a subsequent expansion in verbal language. Movement and physical engagement fostered in DMT stimulated a more general desire to communicate, which naturally extended into the verbal domain. In essence, movement sparked increased use of language.

DMT encourages and activates the body and creates a therapeutic environment where non-verbal and verbal communication continuously intertwine and complement each other. Fan provided a concrete example:

*I believe that when children engage in physical movements, their language expression becomes more diverse and accurate… For example, in the jumping circle game, she not only actively participated but also accurately identified and described the color of the hoop, saying, “this is red, that is blue*.”

Zhang affirmed this view, and stated that both bodily and verbal expression were equally crucial in shaping interaction patterns, whether for expressing closeness or rejection. This interactive process itself was an actual manifestation of the children’s individual development and growth. As Lin summarized, “*Communication often begins with body language and gradually transitions to more complex verbal exchange*,” which highlighted the integrated and developmental nature of non-verbal and verbal communication. In summary, participants perceived their children’s non-verbal and verbal communication not as separate entities, but as a system that developed synergistically through the DMT process.

#### 3.2.4 Category 4: a joyful body and stabilized emotions

Parents saw emotional improvement as a result of DMT. They pointed out that DMT had brought their children happiness and vitality through movement. Pleasant movement helped regulate and stabilize their children’s emotions. After comparing the emotional state of his child at home with that in the treatment video, Lin found that in the video, his child had more stable emotions and faster recovery after an outburst.

*For example, in the first video, my child stands in the center of the classroom and asks the therapist for glass beads. Despite being emotionally excited, she could restrain herself and engage in multiple rounds of conversation with you. If a similar situation occurred at home and my child’s emotions flared up, any attempt to comfort her would be useless. She would only cry and make a scene and roll on the floor*.

Lin continued,

*In the past, I used to stand outside the classroom door every day, ready to rush in and assist the therapist, but even so, we (Lin and the therapist) often felt helpless, and my child was frequently injured as a result*.

Chen emphasized the positive changes in her child’s emotions. She said:

*The duration of my child’s emotional attacks has significantly shortened. Previously, in cognitive and speech therapy classes, her emotional attacks usually lasted for more than 15 min; but now, she can participate in those classes more calmly. Although she sometimes expresses her emotions through movement or sound, such as sitting in a chair and constantly twisting or humming, she can at least cooperate and participate in the treatment*.

Children’s movements exhibited emotional release and joy. Zhang described this by saying, “*When you stood at the other end of the arch bridge and my child ran toward you, I could see joy in her posture*.” Fan similarly observed: “*Now, she often bounces and dances like other children to express happiness.*” Fan also shared touching moments from the session’s video: “*In the video, I capture the ease, joy, and heartfelt happiness in my child’s movement. I have not seen her like this for too long, and perhaps this is the childhood that children should have—pure and simple happiness*.” Hence, DMT can provide a space for children with ASD to fully express their true feelings.

### 3.3 Theme 3: Witnessing an unexpected therapeutic relationship

#### 3.3.1 Category 1: Facilitating participation through movement

Participants frequently reported significant differences between DMT and other intervention methods. They also noted that what happened in DMT differed from their expectations. Specifically, DMT does not rely on any external reinforcement methods but rather stimulates children’s intrinsic interactive potential through the therapist’s movement and verbal encouragement. Owing to its novelty and uniqueness, DMT is highly attractive to children. Consequently, children invest energy and actively participate.

Chen stated:

*I am very grateful that you did not use reinforcement but encouraged her through such words as, “You did a great job.” Recently, I have had to communicate with other therapists because snacks are too often used as teaching reinforcement. We have also tried token systems before, but abandoned them due to poor effectiveness. Therefore, I am particularly grateful to you for not relying on any reinforcement but naturally interacting with my child and supporting her growth.*

Lin also shared his observations. He pointed out that this course (therapy) focused on the child’s strengths, and the therapist’s body movements, gestures, language, and facial expressions were direct and exaggerated, which helped children concentrate better. A few weeks ago, on a Monday afternoon, a parent asked me, “What kind of treatment is this?” I replied, “This is DMT, a course that effectively improves children’s emotions and social problems.” At this point, Lin suddenly turned to me and asked, “*Actually, I am also curious why children are always so happy in your class? In other classrooms, we often hear children crying. To be honest, except for your class, children always cry in treatment.*”

DMT, which does not rely on reinforcement or high pressure as a means, is gradually being accepted by the participants.

#### 3.3.2 Category 2: Acceptance catalyzes children’s expressions

Acceptance is not merely a simple agreement or acknowledgment but a powerful emotional drive that can eliminate interpersonal barriers and defenses (Gabbard and Westen, 2002). In humanistic therapy, unconditional acceptance and empathy are key therapeutic interventions (Bland, 2013). Most participants believed that DMT provided an environment where children experienced acceptance and empathy through movement and were encouraged to express themselves. Parents considered this treatment as a new approach and an opportunity to reflect on their attitudes toward daily caregiving.

Fan stated:

*This is exactly what makes DMT special. You fully accept the child into your emotional world. In this environment, the child finds a space to completely relax their body and mind; ultimately, their growth and development are promoted.*

Zhang’s face showed a curious expression as he said,

*I wonder if it is because you did not apply the pressure of “you should do this, you should do that” when you started working with my child, that she felt comfortable and safe and therefore accepted this course quicker.*

Chen also shared her opinion,

*In fact, during classes at the kindergarten or in other therapy classes, once my child has an emotional outburst, teachers and therapists often adopt a series of educational rules to maintain the classroom order or to continue the treatment process. Even at home, we sometimes adopt this approach under similar circumstances. However, in the water-splashing incident in the video, you did not put my child aside to manage the incident or to blame her, but cleverly transformed this accident into a game opportunity that you and the child could participate in together.*

Through DMT, participants could re-examine and understand the necessity of raising their children with ASD with an open attitude, as well as the importance of actively participating in activities based on acceptance and empathy.

#### 3.3.3 Category 3: Trusting relationships and investment in movement

Participants reported that in DMT, when children experienced increased acceptance and were expressing themselves more through movements, therapeutic relationships were established and defensiveness gradually dissipated. Zhang shared her child’s expectations for the weekly DMT:

*Every Monday afternoon, my child always excitedly asks, “Has Teacher Fan come?”*

Chen also observed,

*My child loves every Monday very much. Even during the break, she rushes to the classroom early to wait for DMT. Moreover, what moved me the most was when my child rode on the neck of the assistant therapist, communicated face-to-face with you, hugged you. This scene showed her great progress in interpersonal relationships and also showed that she had started to trust others*.

### 3.4 Theme 4: Seeing therapy extend into daily life

#### 3.4.1 Category 1: From therapy to daily physical interaction

Multiple moments in the videos surprised and delighted the participants. They experienced these videos as a meaningful opportunity to examine the impact of treatment on their children’s daily behavior changes. Chen shared:

*After watching the videos, I realized that some of the daily interactions between my child and I stem from her therapy experiences. My child’s spontaneous movements have significantly increased. Before going to bed at night, she will actively extend her arms and invite me to lie next to her. In addition, her synchronous behavior is becoming increasingly frequent. For example, when my child walks slowly from one end of the bed to the other along the wall, she will softly request, “Mom, pull me.” Then, we will walk step by step in synchrony, and she always consciously adjusts her pace to keep pace with me. When showering, she will invite me to play water games together. Whenever I submerge beneath the water, she will also submerge with me, and we will happily hold our breath and compete with each other in the water.*

Parents realized DMT promoted bonding at home.

#### 3.4.2 Category 2: Developing self and daily social interaction

DMT enhanced the self-awareness of children with ASD, and this improvement unexpectedly appeared in many daily situations. Compared with their typical “autistic” state of the past, these children now behaved more calmly and were relaxed. Participants reported that their children had gradually adapted to kindergarten life and that their relationships with peers had also improved. In addition, kindergarten teachers reported, during regular communication with parents, that the frequency of their children’s behavioral issues had significantly decreased.

Fan recalled, “*I cannot remember when my child started asking to wear her favorite clothes to DMT classes. My wife felt that our child wanted to show her best self. I guess my child did this in hopes of receiving praise.*”

Zhang also noticed that her child was experiencing similar changes.

*My child likes to showcase herself in front of adults, whether through movement or words. Whenever he hears compliments like “You did a great job!” his desire to show off becomes stronger*. *The most important thing is that my child enjoys this process very much. Although his movements are slightly stiff and his reactions are slightly slow, he is happy and brave to express himself through movement and words*.

Parents noticed that DMT improved their children’s daily relationships. They reported that the therapy made them realize the important role of the body in self-expression and interpersonal interaction. Consequently, parents developed more trust and dependence on DMT.

### 3.5 Theme 5: Reflecting on parenting and family changes

#### 3.5.1 Category 1: Parental self-reflection and emotional awareness

**Figure 10:**
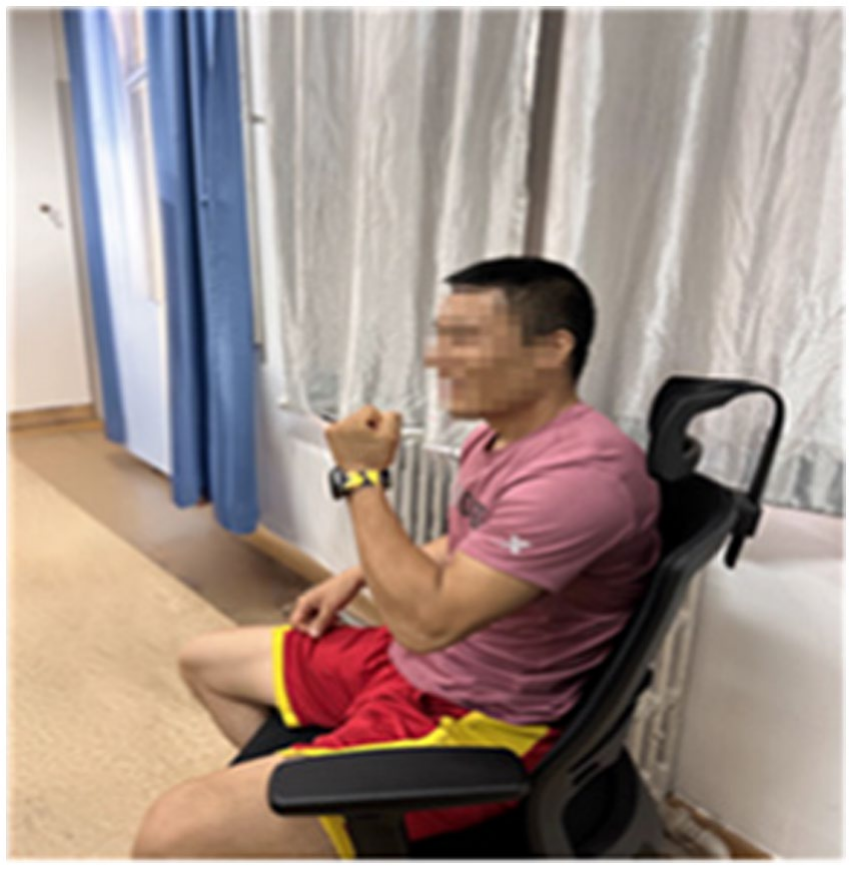
Lin’s bodily response movements.

The act of responding to their children through movement served as a powerful catalyst for parental self-reflection and emotional awareness. For instance, Lin’s movement (see Figure 10), clenching his fists as if gathering all his strength in his palm (Laban Movement Analysis: Increasing Pressure, Strong Weight, Advancing, Sagittal Dimension), was a physical declaration of his determination and resilience. During the interview, Lin said, “*I often perform this movement when excited. It symbolizes growth, strength, and resilience. I hate failure. I want to win. I also hope my child can continue improving and moving forward like me*.” These were traits he consciously hoped to impart to his child.

**Figure 11:**
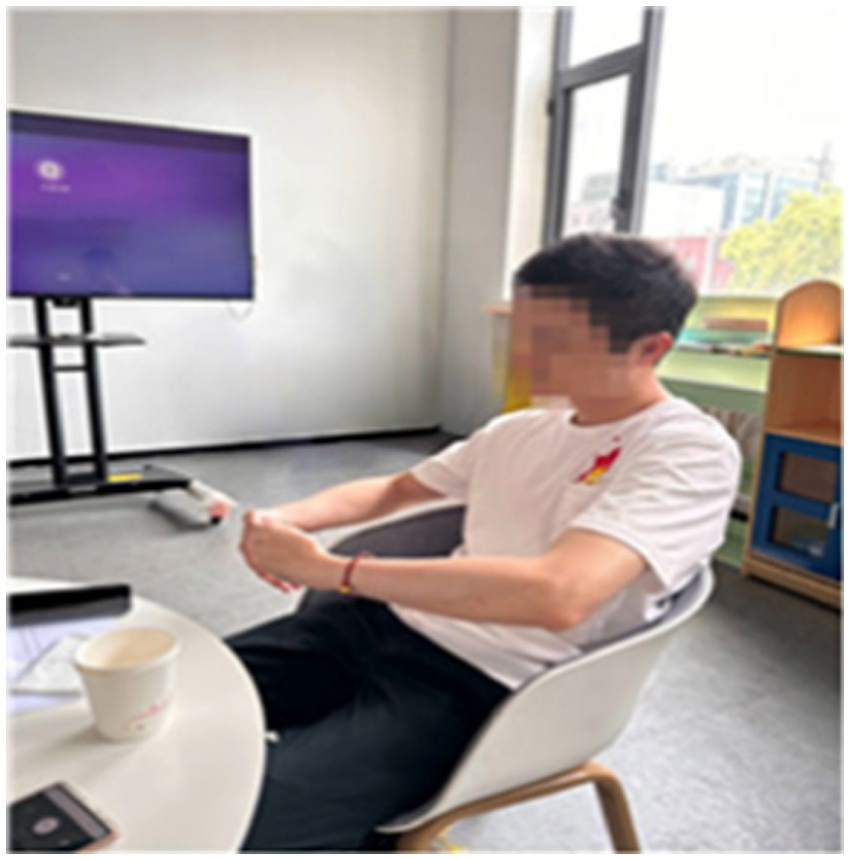
Wang’s bodily response movements.

Wang, Zhang, and Chen also each used unique postures to embrace their children. Wang seemed to be looking at his child, who was in front of him, with a lowered gaze. His movement was characterized as body weight half sinking into the chair, arms closing, and embracing something in a closed form in the horizontal plane (see Figure 11). At that time, Wang explained his actions as follows: “*I never hesitate to express my love to my child. Hugging and conveying emotions is the most natural thing*.”

Chen’s embrace appeared more confident and proactive. Chen’s body naturally leaned forward. This posture’s Laban Movement analysis included direct space, encapsulation, advancing, and horizontal dimension. She explained, “*I chose to squat down and hug her at a height we can see each other. This way, my child would feel safer*.”

**Figure 12:**
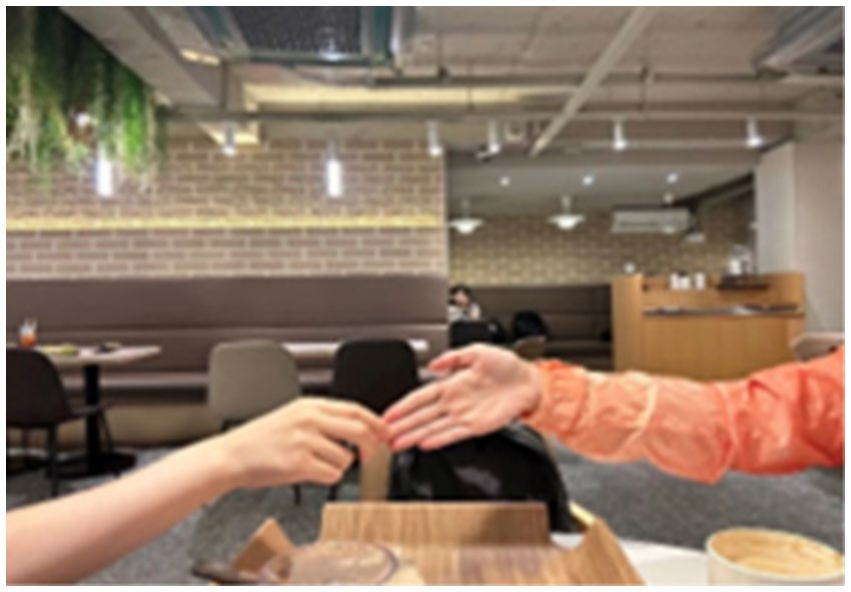
Zhang’s bodily response movements.

Zhang attempted to demonstrate the posture of embracing her child; however, it was difficult to associate her posture with the action of hugging. Zhang talked about her inner feelings. She admitted, “*When I saw my child in the video, I felt he needed a hug. I thought a hug would make him feel at ease. However, I rarely do this kind of thing, so I feel very nervous... no, no, it is too difficult for me*.” Although Zhang’s heart was filled with the desire to hug her child, her body was uneasy due to hesitation and embarrassment. Carrying these conflicting emotions, she mustered up the courage to touch the child’s fingertips lightly but quickly withdrew her hand as if startled (Laban movement analysis: decreasing pressure, light weight, direct space, sagittal dimension) (see Figure 12).

Zhang’s experience most profoundly revealed the role of movement in uncovering hidden emotions. Her attempt to demonstrate a hug was fraught with hesitation and physical unease, directly contradicting her stated desire to comfort her child.

This bodily dissonance triggered a moment of profound self-awareness. She blurted out, “*My child is really like me!*” She realized that her own avoidance behaviors and fear of rejection were mirrored in her child’s difficulties. Hence, movement was instrumental in revealing these hidden emotions and patterns.

This insight led her to articulate a deeper internal conflict regarding her parental identity, express envy of other parents’ closeness alongside her own frustration and impatience, and feel contradictions in her role as a mother.

Thus, these movement experiences transcended mere physical expression; they became a medium for deep emotional and cognitive insights. Through movement, participants engaged in self-confrontation and emotional attunement with their children, and realized it carried and reflected their deepest thoughts, emotions, and attitudes. This newfound awareness fostered their own emotional growth and allowed the parents to observe their children’s movements more sensitively.

#### 3.5.2 Category 2: Transforming parenting and family relationships

DMT catalyzed significant transformations in parenting approaches and family dynamics by fostering interpersonal connections and providing practical guidance. As therapy progressed, parents observed their children with ASD become more proactive and intimate in social interactions.

This newfound proactivity strengthened emotional bonds and improved the family atmosphere that has been dormant for a long time due to ASD. Children began to actively perceive and respond to their parents’ emotions, which marked a subtle but profound shift in relationships.

This was evidenced by increased physical connection and shared joy. Wang shared, “*When I occasionally perform push-ups at home, my child will climb onto my back with great interest*.”

Chen described with passion:

*She will actively take my hand when we go out... Sometimes, she hugs me so tightly that we seem to have become one body... Before going to bed, when I said, “If you love me, then kiss me,” she would respond to my request—behaviors that were unimaginable before*.

Furthermore, participants reported that their children exhibited empathy and initiated shared activities. Wang mentioned that his child started responding to his wife’s tears; Chen described how the three of them would race each other as a family; Fan noted that communication between him and his spouse improved as their child’s condition improved; and Zhang was delighted that her child now actively took her hand and sought her advice, which made their interactions increasingly enjoyable. Thus, DMT strengthened family relationships by bridging the gap between parents and their children through increased physical contact and interactive play, which promoted joyful shared experiences.

This relational transformation was complemented by a shift in parenting paradigms. DMT provided necessary support for families and offered practical guidance for parents on parenting methods. Chen highlighted the transferability of DMT techniques: “*I carefully observed and learned how you interacted with my child... your voice, tone, speed changes, and exaggerated body expressions—I have tried to use them... and they are very effective*.”

DMT served as a unique intervention that provided both transformative relational experiences and concrete, child-centered parenting strategies. It offered participants a deeper understanding of their children’s needs and specific guidance for nurturing their development, which ultimately transformed parenting and family relationships.

#### 3.5.3 Category 3: Recognizing the value and future of DMT

Although limited information is available about DMT in China, and participants were initially hesitant, they made significant discoveries regarding the treatment method after receiving the service. For example, Fan said, “*We were surprised by the daily changes in our child, and simultaneously, it made us, as husband and wife, and the grandparents of our child, more hopeful that DMT could bring even more luck to our child*.”

Zhang, had a similar feeling:

*Even by my picky standards, the overall changes in our child were obvious. He did not stand still nor retreat. This kind of change is unbelievable. What puzzles me even more is that compared to other intensive treatments, even if the child only receives DMT once a week, it can stimulate such positive changes*.

Hence, from the initial hesitation to taking the first step of trying, and subsequently witnessing the actual improvement of their children’s condition, parents accepted and recognized DMT, and were full of hope and confidence in it.

## 4 Discussion and conclusion

Our first research question was, “What experiences do parents of children with ASD gain through their children’s participation in DMT?” The embodiment theory (Shapiro, 2019) explains how bodily experience shapes emotions and cognition. By physically responding to their children’s movements, parents accessed their own emotional worlds and experienced healing insights. Additionally, kinesthetic empathy (Fischman, 2009) helps explain how parents developed emotional attunement by observing or mirroring their children’s bodily expressions. These embodied connections allow for deep relational resonance between the caregiver and child. From a co-regulation perspective (Feldman, 2007), DMT sessions functioned as relational contexts in which children’s emotion regulation was supported through synchronous interactions with therapists; furthermore. These experiences were observed and appreciated by their parents. Emotional shifts and increased self-reflection observed in the parents suggested a transformation in parental-role identity (Burke and Reitzes, 1981). As they observed and engaged with their children differently, their self-perception as parents evolved in meaningful ways. Symbolic interactionism (Blumer, 1986) provides a useful lens to understand how the parents came to interpret their children’s non-verbal expressions as meaningful communication, shifting everyday interactions toward greater mutual understanding.

In Theme 2 (Observing Embodied Communication and Emotional Growth), participants observed that the therapist accurately captured their children’s movement during the treatment process and provided support in various physical, cognitive, and emotional aspects. Participants believed that these non-verbal expressions effectively compensated for their children’s language deficiencies (Chaiklin and Schmais, 1979). They stressed that movement and language both supported communication in children with ASD, as movement was inherently meaningful and communicative (Sheets-Johnstone, 2011). Our results reveal the following therapeutic mechanisms.

First, DMT is similar to a magnifying glass, and presents a child’s inner world through movement. Participating parents explicitly stated their understanding that DMT was a psychological therapy method fundamentally different from physical therapy or rehabilitation aimed solely at improving motor coordination and enhancing physical fitness. Zhang praised DMT and stated, “*Paying attention to and protecting children’s inner health is more important than teaching them skills*.” This was consistent with previous results that found movement was similar to a mirror and able to reflect children’s inner thoughts and emotional state (Lewis et al., 2000). The therapist provides attunement, feedback, and interventions based on their understanding and analysis of the children’s movement.

Second, dance/movement is as important as language, and reflects the fundamental unity of body and mind. As Levin (1999) stated: “We must use the tool of the body to understand external *reality. In actual life, there is no strict boundary between the body and the mind; they together help us understand the world*.” This foundational perspective confirms that non-verbal expression is a primary mode of human cognition and connection in its own right, rather than merely supplementary to verbal language. As Chen pointed out, “*In early childhood, the development of body language often precedes spoken language. Especially for preschool children, their body expressions convey rich and direct information*.” Furthermore, Lin shared his profound insights on the importance of non-verbal elements in children’s communication. He emphasized that in interpersonal interactions, lack of eye contact, inadequate language expression, stiff posture, and sluggish facial expressions were all important factors in effective communication.

Third, movement is an important tool for expressing relationships, and its role cannot be replaced by verbal language. As participating parents gained a deeper understanding of the function of movement, they re-examined the relationship between language and non-verbal information, and understood that the meaning of non-verbal actions was rooted in the universality of bodily experience (Moore and Yamamoto, 2012). Accordingly, they considered how to further sensitively capture and understand their children’s non-verbal information in daily life. In Fan’s discovery, the richer and more active his child’s movement was, the more active and vivid the child’s language expression became. Zhang also understood her child’s behaviors better: “*Refusal is a way for my child to express his own needs and emotions in interaction, which in itself indicates that my child is in a certain relationship and has invested a certain amount of emotion and energy in it*” (Adler, 1970). Parents began paying closer attention to their children’s non-verbal behaviors.

Fourth, emphasis on unconditional acceptance and empathy is a unique feature of DMT. In Theme 3, participants understood the concept of movement and gained a deeper understanding of the significant differences between the practices of DMT and their original beliefs. Prominently, parents observed DMT as a “safe outlet for children’s expression.” They observed that the dance/movement therapist never forced their children to perform specific behaviors; instead, they always provided them with wholehearted acceptance, understanding, and genuine responses, whether through verbal or non-verbal means. Chase and Whitehouse highlighted that DMT was essentially a relational therapy approach; hence, establishing therapeutic relationships is crucial for its development and advancement (Levy, 1988). In some situations, the therapist is similar to a mirror for the children, and reflects their body rhythms and movement patterns through non-verbal expressions. Emotional interactions naturally occur in this process (Chaiklin and Schmais, 1993). “*I can see you. I like how you are right now. You can be yourself*.” (Devereaux, 2012). These messages are conveyed through physical actions. Participants reported that the therapist’s affirmation and encouragement significantly enhanced their children’s trust and dependence on the therapist in the therapeutic relationship. This mutual bond encouraged children’s deeper engagement.

In DMT, acceptance and empathy can build a stable two-way relationship between the therapist and the children, and may become a powerful engine for reshaping parent–child interactive patterns. Understanding these treatment mechanisms can help parents adjust their parenting style and emotional engagement, reduce reprimands, and eliminate misunderstandings. Specifically, changes in parents can potentially cultivate empathy and improve interpersonal communication skills in their children with ASD, which is consistent with previous research. Parents’ empathy may be mirrored by their children (Grusec and Hastings, 2014). Meanwhile, warmth and tolerance from parents can enhance children’s interpersonal connections and encourage their emotional responses to others (Hoffman, 2000). In addition, increased emotional support helps children understand their own and others’ emotional needs, which can promote emotion regulation (Malti et al., 2013). Therefore, being inclusive and empathetic as parents can positively impact children’s growth.

These findings were consistent with the global trend toward inclusive and person-centered approaches in autism intervention, which value the voices of children and families and build on their unique strengths (Pellicano et al., 2018). DMT focuses on embodied expression and relational engagement and offers a therapeutic pathway that complements existing behavioral or skill-based interventions with a further holistic, family-centered orientation.

These findings deepen our understanding of parental experiences with DMT and resonate with movements in autism research that emphasize inclusivity and person-centered support. These perspectives provide a bridge to the conclusion, where we highlight the broader implications of the study for practice and future research.

Our second research question was, “What significance does dance movement therapy (DMT) hold for parents of children with ASD?” Theme 1 clearly reflected how the parents of children with ASD viewed DMT at the beginning of treatment and how their views changed during the treatment process. When participants first encountered DMT for their children with ASD, their cognition was greatly restricted due to the limited information in China. Many thought DMT was merely a dance class. This indicates that without direct clinical experience of DMT, parents would lack understanding and information about it, and DMT is still in its early stages academically in China.

However, despite limited information and understanding of DMT among Chinese parents, participating parents still demonstrated a positive attitude and open-mindedness toward attempting new therapeutic interventions for their children. This reflects these parents’ urgent desire to find diverse and effective treatment methods for their children’s ASD symptoms and belief that their children’s treatment and recovery are their responsibility. This sense of urgency may stem from the strong sense of mission toward the “parental role” in Chinese family culture. According to Fan and Ko’s (2023, 2025) studies, parents often subconsciously believe that their children’s ASD is their fault. When they feel a deep level of self-blame, their sense of guilt and selfless behavior toward their children becomes excessive. This prompts them to explore all possible ways to improve their children’s health and wellbeing (Myers et al., 2009), which may explain participants’ willingness to take risks, try new methods, and challenge themselves.

As the treatment progressed, parents’ understanding of DMT also underwent significant changes. Watching session videos changed their views. When witnessing small but positive changes in their children, participants developed a sense of recognition and gratitude toward DMT; furthermore, their expectations that their children would continue to change and develop also increased accordingly.

Their children’s verbal expressions included, “*I am a little donkey, Mom is a donkey mom,*” playful conversations with the therapist through association, such as “*This is skin color*,” and reaction when expressing dissatisfaction, “*You go find Mom then, hmph!*” These results were consistent with Fan and Ko’s (2023) findings, and provide additional evidence that the emphasis on interrelationships and quality of interaction between therapists and children in DMT can stimulate expressions and responses of children with ASD. Parents found that DMT enhanced their children’s range of expression and emotional control.

In addition, most parents felt that their children exhibited many positive changes in emotional expression after participating in DMT, and significantly improved in their emotion-regulation abilities. Lin stated that after receiving DMT, the frequency, duration, and intensity of his child’s emotional attacks significantly diminished. Furthermore, Chen’s child’s emotional expression became more stable. These positive changes were not only observed during DMT but also confirmed in their other daily environments outside the treatment room. Moreover, Zhang and Fan found that their children’s mood had become more optimistic during the DMT process. In fact, sensory stimulation and physical contact may have lifelong impacts on a child’s development, as these interactions can influence the brain’s internal connectivity and activation patterns (Bloom, 2006; Hammond, 2009). Therefore, DMT may multidimensionally influence children’s development via movement and non-verbal interaction. Literature indicates that DMT can enhance children’s emotional recognition abilities and prompt the use of internal emotional coping resources and appropriate external expression of emotions (Fischer and Chaiklin, 1993; Mohan, 2020; Scatozza et al., 2023).

Theme 5 explores how the parents gradually and fundamentally changed their understanding of their children by observing their session videos. In the treatment sessions, the interaction between the therapist and the children was not one-directional; rather, it was a dynamic relationship of mutual influence. Hadjikhani (2007) found that DMT expanded the movement patterns of children with ASD in interactions and significantly improved their social cognitive abilities. Participant Chen described, “*My child and I coordinated our movements together and she consciously followed my movements. We played breath-holding games in the water while squatting or standing together.*” This participant realized that this synchronized breathing between mother and daughter reflected the interactive experience in DMT, and reported, “*After watching the recording, I realized that our daily interactions were actually repeating the behaviors in the DMT* class.” These experiences helped parents rediscover DMT’s therapeutic value. The firsthand accounts from the parents provide strong empirical validation for the theoretical frameworks mentioned: embodied connection (Shapiro, 2019), kinesthetic empathy (Fischman, 2009), and synchronized regulation (Feldman, 2007). These occurrences were observed by the parents and actively extended into their daily interactions, which ultimately lead to profound changes in their understanding and perception of DMT.

In Theme 5, parents generally reflected that DMT directly affected the children and also extended its effects to the whole family, as a change in one member (the child) inevitably influenced and reshaped the patterns of interaction for all. This process began as children spontaneously started to seek emotional connection with their parents through intimate physical contact, such as holding hands and hugging. Furthermore, the children underwent significant emotional changes and gradually demonstrated empathy toward their parents’ emotions. Wang mentioned that when her child saw her crying, he would cry along with her. This phenomenon transcends simple imitation; it represents the development of neural resonance, the capacity to mirror and empathize the affective state of another (Feldman, 2017). This emotional resonance, rooted in the mutual attention and love between parent and child, provides crucial emotional support for both parties, and establishes a positive feedback loop of co-regulation.

Although the findings of our in-depth study provide valuable insights into parents’ experiences with DMT, its generalizability may be limited. First, the small sample of five participants were recruited from one region and had prior engagement with the researcher, which could have potentially influenced their responses. Future studies should involve further diverse participants and explore DMT’s long-term effects from the perspectives of multiple stakeholders. Examining support groups for parents and cross-cultural comparisons may further reveal DMT’s adaptability and global relevance.

Importantly, this study highlights that parents recognized their children’s challenges and also their strengths, such as creativity, persistence, and unique ways of expressing themselves, through DMT. This strengths-based perspective is central to advancing inclusive practices that move beyond deficit-oriented models of autism.

Finally, this study deepened the understanding and recognition of the unique value of DMT among Chinese parents of children with ASD. Furthermore, it increased the possibility of introducing DMT into the clinical-service system in China. Specifically, this study provides diverse and high-quality rehabilitation options for Chinese special-needs children and their families. At the macro level, this study fully considers the developing stage of DMT in China. Combined with profound reflections on Chinese cultural characteristics, it lays a solid foundation for the localization and transformation of DMT in China.

## Data Availability

The raw data supporting the conclusions of this article will be made available by the authors, without undue reservation.
